# Unveiling psychobiological correlates in primary Sjögren’s syndrome: a machine learning approach to determinants of disease burden

**DOI:** 10.1192/j.eurpsy.2026.11805

**Published:** 2026-07-31

**Authors:** L. Módis, A. Matuz, Z. Aradi, I. F. Horváth, A. Szántó, A. Bugán

**Affiliations:** 1 Department of Behavioural Sciences, Faculty of Medicine, University of Debrecen, Debrecen; 2 Department of Behavioural Sciences, Medical School; 3 Szentágothai Research Centre, University of Pécs, Pécs; 4 Division of Clinical Immunology, Department of Internal Medicine, Faculty of Medicine, University of Debrecen, Debrecen, Hungary

## Abstract

**Introduction:**

Besides primary Sjögren’s syndrome (pSS) is generally assessed through biological markers, growing evidence suggests that psychological and social factors-such as anxiety, depression, personality traits, and social support-may also play a role in disease burden. Relative contribution of these biopsychosocial dimensions to disease activity in pSS, however, has not been quantitatively compared.

**Objectives:**

This study aimed to evaluate the predictive weight of different factors in determining both objective and subjective disease burden using machine learning (ML) models.

**Methods:**

117 pSS patients, whose biological (blood cell counts, complement activity, IgG, RF, SSA, SSB), psychological (personality traits, depression, anxiety, basic self-esteem assessed via self-reported questionnaires), and social (socioeconomic status and social support) measures were collected in a composite database. Outcome variables were SSA/SSB autoantibodies and EULAR Sjögren Syndrome Patient Reported Index (ESSPRI), as indicators of biological and perceived disease burden, respectively. Three machine learning algorithms were trained to predict outcome variables, first by each measure category, then on the entire set of predictor variables. Permutation feature importance was used to assess the importance of the predictors. The five most important predictors were selected for all target outcomes.

**Results:**

Concerning autoantibodies, the model performed best with biological input only, in the case of ESSPRI, the complete dataset gave the best performance. Trait anxiety was selected as important negative predictor of both autoantibodies. Besides, biological measures (IgG, RF, platelet count) and age were among the five most important features. State anxiety and temperament trait ‘Fatigability’ were important positive predictors of ESSPRI, while character trait ‘Pure-hearted conscience’, IgG and RF were important negative predictors.

**Conclusions:**

Unexpected psychobiological correlations, like trait anxiety and IgG/RF as negative predictors of autoantibodies and ESSPRI, respectively, suggest different (immunobiological and psychosomatic) disease mechanisms and symptom burden. Importance of psychological factors in estimating disease burden may pave the way toward novel, more sensitive diagnostic tools and therapeutic methods and better understanding of pathomechanisms of pSS.

**Disclosure of Interest:**

None Declared

